# 

**Published:** 1882-12

**Authors:** 


### HALL'S JOURNAL OF HEALTH AND MISCELLANY.

MONTHLY.

E. H. GIBBS, A. M., M. D., EDITOR.

FOUNDED IN 1854.

### SEVERAL GOOD OFFERS.

THIS number completes the Twenty-Ninth year of the "JOURNAL OF HEALTH." During three hundred and forty-eight consecutive months it has been sent out to its readers, its pages replete with suggestions for the care of the health and the best methods of avoiding most of the diseases and accidents that come from a want of this knowledge; but although the JOURNAL has attained a popularity not enjoyed by any other similar publication, and its opinions are much quoted by newspapers throughout the country; and although every intelligent person admits that no knowledge is dear at any price, that promises to promote health, and secure length of life; the discouraging fact still remains that the majority prefer to invest ten dollars in trashy reading, to investing two dollars in a monthly publication which at the end of the year makes one of the most useful volumes that a person can have as a book of reference, in matters relating to health; particularly in families, where such information is often required.

The present circulation of the "JOURNAL" is nearly seventeen thousand copies per month; and it would be a low estimate to put the number of its readers at one hundred thousand each month. Occasionally an article, perhaps on Diphtheria, appearing in the JOURNAL at a time when that terrible disease is prevalent, will be quoted by a thousand newspapers in the United States and Canada; thus calling the attention of a million readers to the JOURNAL OF HEALTH; but what is a million in comparison with the fifty millions who should receive such instruction, and many of whose lives are sacrificed for the want of such advice as the JOURNAL gives? Now our ambition is to attain for the JOURNAL a circulation of ONE HUNDRED THOUSAND copies each month; and to the accomplishment of this object we will make the following concessions, until further notice. Every NEW subscriber who sends us ONE DOLLAR will receive the Journal of Health one year. All public Institutions, Schools, Societies, Associations, Libraries, Reading Rooms, or Organizations whatever—not already subscribers—that will send us 50 CENTS, which is barely sufficient to cover the cost of the paper before it is printed, will receive the Journal of Health one year.

We call attention to our Table of Contents for the year 1882, published in this number of the Journal. It is intended to make the Journal during 1883 better than it has ever been before, since there will be a far greater number of subjects discussed, and the volume which the twelve monthly numbers will make at the end of the year will be larger and handsomer than any that has preceded it.

If any person who preserves the twelve numbers of the Journal for 1883 will send them to us at the end of the year with seventy-five cents we will have them handsomely bound in cloth and returned postage paid. We will take Canada money for Canadian subscriptions.

